# Nisin-based therapy: a realistic and eco-friendly biocontrol strategy to contrast *Xylella fastidiosa* subsp. *pauca* infections *in planta*

**DOI:** 10.3389/fmicb.2024.1406672

**Published:** 2024-05-15

**Authors:** Miloud Sabri, Kaoutar El Handi, Franco Valentini, Angelo De Stradis, Orges Cara, Cosima Damiana Calvano, Mariachiara Bianco, Antonio Trani, Toufic Elbeaino

**Affiliations:** ^1^International Centre for Advanced Mediterranean Agronomic Studies (CIHEAM of Bari), Bari, Italy; ^2^National Research Council of Italy (CNR), Institute for Sustainable Plant Protection (IPSP), University of Bari, Bari, Italy; ^3^Department of Soil, Plant and Food Science, University of Bari, Bari, Italy; ^4^Department of Chemistry, University of Bari, Bari, Italy; ^5^National Research Council of Italy (CNR), Institute for Sustainable Plant Protection (IPSP), Naples, Italy

**Keywords:** bacteria, antimicrobial peptide, v-qPCR, electron microscopy, RPLC-ESI-MS/MS, biocontrol

## Abstract

The lack of sustainable strategies for combating *Xylella fastidiosa* (*Xf*) highlights the pressing need for novel practical antibacterial tools. In this study, *Lactococcus lactis* subsp. *lactis* strain ATCC 11454 (*L. lactis*), known for its production of nisin A, was *in vitro* tested against *Xf* subsp. *pauca*. Preliminary investigations showed that nisin A was involved in a strong antagonistic activity exhibited by *L. lactis* against *Xf*. Thus, the efficacy of nisin A was comprehensively assessed through a combination of *in vitro* and *in planta* experiments. *In vitro* investigations employing viable-quantitative PCR, spot assay, turbidity reduction assay, fluorescence microscopy, and transmission electron microscopy demonstrated nisin’s robust bactericidal effect on *Xf* at a minimal lethal concentration of 0.6 mg/mL. Moreover, results from fluorescence and transmission electron microscopies indicated that nisin directly and rapidly interacts with the membranes of *Xf* cells, leading to the destruction of bacterial cells in few minutes. In *in planta* tests, nisin also demonstrated the ability to tackle *Xf* infections within *Nicotiana benthamiana* plants that remained asymptomatic 74 days post inoculation. Furthermore, RPLC-ESI-MS/MS analyses showed that nisin translocated to all parts of the plants and remains intact for up to 9 days. For the first time, this study underscores the nisin-based strategy as a realistic and eco-friendly approach to be further investigated against *Xf* infections in the field.

## 1 Introduction

*Xylella fastidiosa* (*Xf*) is a Gram-negative bacterium that continues to inflict substantial losses to various important crops throughout the world, including coffee, citrus, grapevine, olive, and almond. In Italy, following the initial detection of *Xf* subsp. *pauca* in Apulia region in 2013, it has emerged as the predominant threat to olive trees in the country (Velasco-Amo et al., 2023). *Xylella fastidiosa* infections have led to a significant decline in olive crop yields, with approximately 5 million trees infected or dead, representing losses equivalent to around 10% of the Italian olive production (White et al., 2020). Beyond the direct economic implications, *Xf* diseases have also exerted noteworthy societal impacts (Ali et al., 2021). The challenges arise from the scarcity of effective bactericides and the inherent difficulty in accessing the vascular system of the plant, where the pathogen establishes itself (Ali et al., 2021). Although, in rare cases, some treatments promise to reduce the population level of the bacteria in infected plants, the challenge persists due to the lack of sustainability of these treatments (Scortichini et al., 2021). This underscores the pressing need for novel biocontrol strategies in *Xf* disease management. In response to these challenges, environmentally friendly options and sustainable strategies that conform to European natural trends are needed. Bacteriocins, which are polypeptides or precursor polypeptides with bacteriostatic or bactericidal activity produced by certain bacteria in the metabolic process through ribosome synthesis mechanism, have become a research hotspot for new antibacterial agents due to their low cost, complex and diverse bactericidal mechanisms of action (Simons et al., 2020). Among bacteriocins, nisin A (hereafter named nisin) represents the most prominent and widely applied natural bacteriocin with high antibacterial activity against a wide range of Gram-positive bacteria (Field et al., 2023).

Nisin, a member of the class I bacteriocins referred to as lantibiotics, is produced by certain *Lactococcus lactis* subsp. *lactis* strains (*L. lactis*) (Field et al., 2023). It is distinguished by a cationic, polycyclic, thermostable peptide structure with a molecular weight of 3.4 kDa, and a broad spectrum of antibacterial activity primarily targeting Gram-positive bacteria (Małaczewska and Kaczorek-Łukowska, 2021). The antibacterial activity of nisin against Gram-positive and -negative bacteria stems from its triple mode of action, which includes binding to lipid II, a crucial precursor in peptidoglycan biosynthesis, formation of pores within the cell membrane made up of lipid II, and directly degrading target cell DNA (Field et al., 2023). By binding to lipid II and forming pores in the membrane, nisin can cause the efflux of cellular constituents and inhibit cell wall biosynthesis. However, the activity of nisin against Gram-negative bacteria could in some cases be much lower than that against Gram-positive bacteria, mainly because lipid II is located at the inner membrane, and the rather impermeable outer membrane in Gram-negative bacteria prevents nisin from reaching lipid II (Li et al., 2018). Leveraging its potent antimicrobial properties, minimal propensity to induce bacterial resistance, and low cellular cytotoxicity at antimicrobial concentrations, nisin has proven highly effective against drug-resistant bacteria (Barbosa et al., 2021) and has found successful application in the field of food preservation (Ibarra-Sánchez et al., 2020). These characteristics in conjunction with its generally regarded as safe (GRAS) status and its stability under various conditions, have contributed to the worldwide success of nisin as a natural food preservative (E234) (Field et al., 2023). These advantages collectively make nisin a potential antibacterial agent for the control of bacterial plant diseases, potentially complementing existing biocontrol agents in the pursuit of integrative, sustainable, and environmentally friendly agriculture.

Despite the recognized importance of nisin, it has not been yet examined for its efficacy against plant pathogenic bacteria. This study addresses this gap by presenting, for the first time, an in-depth investigation into the antagonistic effect of *L. lactis* and its well-established bacteriocin, nisin, against *Xf* subsp. *pauca* both *in vitro* and *in planta*.

## 2 Materials and methods

### 2.1 Bacterial and growth conditions

*Xylella fastidiosa* subsp. *pauca* strain A0PT1, isolated from olive trees affected by the olive quick decline syndrome (OQDS) in southern Italy, Apulia region, was used in all experiments. The strain was stored in Pierce disease broth (PD2) (Davis et al., 1980) supplemented with glycerol (50%) and maintained at −80°C. When needed, aliquots were cultured in buffered charcoal yeast extract (BCYE) (Wells et al., 1981) agar plates and grown at 28°C for 10 to15 days. Cell suspensions were prepared using phosphate buffered saline (PBS, pH 7.4, 0.01 M) and adjusted to 10^8^ CFU/mL (OD600 ≅ 0.32) (El Handi et al., 2022). *L. lactis* subsp. *lactis* ATCC 11454 was grown on YPGA plates (5.0 g/L yeast extract, 5.0 g/L peptone, 10.0 g/L glucose, and 15.0 g/L agar) for 2 days at 28°C.

Nisin (ITSISLCTPGCKTGALMGCNMKTATCHCSIHVSK), extracted and purified from *L. lactis*, was purchased from Sigma-Aldrich (Merck KGaA, Darmstadt, Germany). According to the manufacturer, the formulation contains 2.5% (w/w) pure nisin with potency ≥900 IU/mg. Lyophilized nisin was solubilized in sterile Milli-Q water to a stock concentration of 12.5 mg/mL and filter sterilized through a 0.22 μm nylon Acrodisc^®^ syringe filter (Merck, Rome, Italy).

### 2.2 *In-vitro* investigations for antagonism between *Lactococcus lactis* subsp. *lactis* and *Xylella fastidiosa*

The antagonistic effect of *L. lactis* subsp. *lactis* ATCC 11454 against *Xf* subsp. *pauca* was investigated as follows: four drops of *Xf* suspension (10^8^ CFU/mL), each containing 30 μL, were positioned at the upper portion of the BCYE agar plates, with approximately 1.5 cm between them. These drops were allowed to slowly descend to the opposite side, forming four parallel rows of *Xf* cultures. Subsequently, two drops (10 μL each) of *Lactococcus lactis* suspension (10^8^ CFU/mL), prepared in PBS, were administered to the center of the rows of *Xf* cultures after drying under the laminar flow hood. PBS served as the negative control. Following an incubation period of 10 days at 28°C, the antagonistic activity of *Lactococcus lactis* was perceived as an inhibition zone of *Xf* growth, which was measured using a digital caliper. This experiment was conducted in triplicate for reliability.

### 2.3 Characterization of proteins secreted by *Lactococcus lactis* challenged by *Xylella fastidiosa*

Reversed-phase liquid chromatography (RPLC) coupled with electrospray (ESI) and tandem mass spectrometry (MS/MS) was used to explore the proteins involved in the inhibitory effect induced by *L. lactis* against *Xf*. For inhibition effect examination, two pieces of culture medium from the inhibition zone were cut and subjected to extraction with 200 μL of 10 mM Tri-HCl for an easy protein/peptide recovery. In addition, 100 μL of *L. lactis* suspension (10^8^ CFU/mL) was challenged by 100 μL of *Xf* suspension (10^8^ CFU/mL) in 2 mL of YPG liquid medium for 24 h at 28°C. After incubation, the mixture was passed through a 0.22 μm pore filter and subjected to RPLC-ESI-MS/MS.

For analysis, water, acetonitrile, and formic acid LC-MS grade were purchased from Merck (Milan, Italy). Five microliters of the extract or standard solutions were injected in the UHPLC Ultimate 3000 system (Dionex Thermo Fisher Scientific, Italy) coupled to HESI (heated electrospray ionization) interface and VelosPro mass spectrometer (Thermo Scientific, Waltham, MA, United States) equipped with a double linear trap mass analyzer. The reversed-phase (RP) chromatographic separations were accomplished at 40°C using a Phenomenex Aeris WIDEPORE 200 Å C18 column (250 × 2.1 mm, 3.6 μm) equipped with Phenomenex AJO 8783 WIDEPORE C18 (2 × 2.1 mm ID) security guard cartridge and a mobile phase based on H_2_O (solvent A) and ACN (solvent B) both containing 0.1% of formic acid. Specifically, the gradient used during each chromatographic run, at a flow rate of 0.200 mL/min, was the following: 0–3 min linear from 5 to 20% solvent B; 3–5 min 20% solvent B; 5–12 min linear from 20 to 85% (v/v) solvent B; 12–16 min isocratic at 85% of solvent B; 16–17 min back to the initial composition, followed by 5 min equilibration time. Mass spectrometry analyses were carried out in full scan and in selected reaction monitored (SRM) transitions mode in positive polarity or in targeted MS/MS mode.

### 2.4 Nisin minimal lethal concentration

Given the unique characteristics of *Xf* (slow growing propriety), an initial *in vitro* screening was performed to determine an approximate initial concentration of nisin suitable for subsequent challenges to *Xf* cells. This preliminary screening was assessed by a contact test coupled with viable-quantitative PCR (v-qPCR) using the PMAxx^™^ (Biotium, Rome, Italy) (Baró et al., 2020). PMAxx^™^ is a photo-reactive dye that forms a covalent bond with disrupted DNA, rendering it un-amplifiable by PCR. This unique feature makes PMAxx^™^ extremely useful in selective detection of bacterial cells with intact plasma membranes by qPCR. Briefly, 50 μL of *Xf* cells with OD at 600 nm of 0.32 measured by NanoDrop^™^ One/OneC Microvolume UV–Vis Spectrophotometer (Thermo Fisher Scientific), were treated with 50 μL of nisin at 6, 3, 1.5, 1, 0.8, 0.6, 0.4, 0.2, 0.1, and 0.05 mg/mL. Consequently, series of dilutions of *Xf* ranging from OD = 0.32 to OD = 0.02 were employed as controls, and samples were incubated for 3 h at 28°C. After incubation, samples were treated with PMAxx^™^ at a final concentration of 7.5 μM, incubated in the dark at room temperature for 8 min, and followed by a 15 min photoactivation step using the PMA-Lite^™^ LED Photolysis Device. Genomic DNA of all samples was extracted following the CTAB protocol (Burbank and Ortega, 2018). For analysis, a TaqMan-based qPCR assay was carried out in a thermocycler apparatus (Bio-Rad CFX96, BioRad, Milan, Italy), utilizing specific primers *Xf*-F: 5′-CACGGCTGGTAACGGAAGA-3′ and *Xf*-R: 5′-GGGTTGCGTGGTGAAATCAAG-3′, along with the probe *Xf*-Prb: 5′-6FAM-TCGCATCCCGTGGCTCAGTCC-BHQ-1-3′ (Burbank and Ortega, 2018).

### 2.5 Spot assay

To corroborate the findings derived from the v-qPCR assay pertaining to the lethal concentrations of nisin against *Xf*, a spot assay was conducted as follows: *Xf* subsp. *pauca* was cultured at 28°C on BCYE agar plates for up to 10 days. Then, the cultures were suspended in PBS and 30 μL of bacterial suspension (OD_600_ = 0.32) were mixed with 30 μL of nisin at 3, 1.5, 1, 0.8, and 0.6 mg/mL. Subsequently, the mixtures were spotted onto the surface of the BCYE agar plates and allowed to air-dry within the laminar flow hood. Negative controls consisted of PBS, while positive controls involved 30 μL of *Xf* mixed with 30 μL of PBS (Mourou et al., 2022). Growth inhibition was assessed 10 days post-incubation at 28°C, checked by the level of *Xf* growth. The experiment was conducted with three independent replicates.

### 2.6 Turbidity reduction assay

The effect of the minimal lethal concentration (MLC) of nisin on *Xf* cells in liquid medium was assessed by quantifying the degree of turbidity at OD_600_ in both nisin-treated and untreated *Xf* suspensions over 3 h. Briefly, a 50 μL aliquot of *Xf* suspension in PD2 (Pierce disease 2 media) was mixed with 50 μL of nisin at 0.6 mg/mL, resulting in a final turbidity of *Xf* with an OD_600_ of 0.41. Untreated control involved 50 μL of *Xf* mixed with 50 μL of PBS with a final turbidity of 0.41 at OD_600_. Bacterial turbidity was assessed by tracking the OD_600_ using the NanoDrop^™^ One/OneC Microvolume UV–Vis Spectrophotometer at 0 min, 1 h, 2 h, and 3 h of incubation at 28°C. The experiment was conducted twice, with three replicates for each treatment.

### 2.7 Fluorescence microscopy

Fluorescence microscopy (FM) provides an accurate assessment methodology for the bacterial lysis process by enumerating the populations of intact and permeable cells at various time intervals. In this context, 100 μL *Xf* suspension (OD_600_ = 0.32) was mixed with 100 μL of nisin at its MLC (0.6 mg/mL) and incubated at room temperature for 3 h. Untreated control consisted of bacterial suspension treated with sterile distilled water. To assess the viability of *Xf* cells exposed to nisin, the LIVE/DEAD^®^ BacLight^™^ viability kit (provided by Molecular Probes) was employed. This kit contains two nucleic acid dyes, SYTO 9 and propidium iodide (PI), which enable the differentiation of live cells with intact plasma membranes (visualized in the green channel) from bacteria that have succumbed to membrane compromise due to nisin activity (visible in the red channel). Photomicrographs were captured at 15 min, 30 min, 1 h, 2 h and 3 h post-incubation, using a Nikon E800 microscope equipped with fluorescein isothiocyanate (480/30 excitation filter, DM505 dichroic mirror, 535/40 emission filter) and tetramethyl rhodamine isothiocyanate (546/10 excitation filter, DM575 dichroic mirror, 590 emission filter) fluorescence filter sets.

### 2.8 Transmission electron microscopy

To scrutinize the antibacterial properties of nisin against *Xf* cells, a suspension of *Xf* (OD_600_ = 0.32) was exposed to nisin at 0.6 mg/mL for a duration of 3 h at room temperature. Representative images were taken after 3 h via transmission electron microscopy (TEM) (FEI MORGAGNI 282D, United States) using the dip method. Briefly, carbon-coated copper/rhodium grids were immersed in both the nisin-treated and untreated bacterial suspensions for 5 min, followed by a rinse with 200 μL of distilled water. To achieve negative staining, the grids were floated on 200 μL of a 0.5% w/v UA-zero EM stain solution (Agar-Scientific Ltd., Stansted, United Kingdom), and observed under an accelerating voltage of 80 kV.

### 2.9 Spectrum of action

The nisin’s spectrum of action at MLC (0.6 mg/mL) was assessed by targeting various pathogenic and beneficial plant bacteria using the spot assay. Therefore, 16 phytopathogenic bacterial isolates, including three different isolates of *Xf* subsp. *pauca*, together with one epiphytic (*Pantoea agglomerans*) and three endophytic bacteria (*Bacillus subtilis*, *Paenibacillus rigui*, and *Bacillus pumilus*) isolated from olive trees, which are reported in the literature to exhibit antibacterial activity against *Xf* subsp. *pauca* (Mourou et al., 2022), were examined (Table 1). For *Xf* isolates, spot tests were carried out on BCYE agar plates as described earlier. For other bacteria, spot tests were performed on YPGA plates as follows: bacterial isolates were cultured at 28°C on YPGA plates for up to 2 days. Then, the cultures were suspended in sterile distilled water and 200 μL of bacterial suspension (OD_600_ = 0.2) were mixed with 6 mL of YPG soft agar (i.e., YPG supplemented with 0.7% agar), poured into YPGA plates, and allowed to dry. Subsequently, drops of 10 μL of nisin were spotted onto the surface of the plates. Spots were dried at room temperature and the plates cultured for up to 2 days at 28°C. The presence of a clear zone in the spot area was indicative of bacterial susceptibility to nisin. The experiment was replicated three times for validation.

**Table 1:** Bacterial strains used for determining the nisin’s spectrum of action.

Species	Isolate	Host plant	Origins
*Bacillus subtilis*	L39^a^	*Olea europaea*	Italy
*Paenibacillus rigui*	S55^a^	*Olea europaea*	Italy
*Bacillus pumilus*	L36^a^	*Olea europaea*	Italy
*Pantoea agglomerans*	BA69^a^	*Nerium oleander*	Italy
*Xylella fastidiosa* subsp. *pauca*	IAMB A0PT1^a^	*Olea europaea*	Italy
*Xylella fastidiosa* subsp. *pauca*	IAMB OR1^a^	*Oleander nerium*	Italy
*Xylella fastidiosa* subsp. *pauca*	IAMB B3^a^	*Polygala myrtifolia*	Italy
*Xanthomonas albilineans*	CFBP 1943	*—*	Burkina Faso
*Xanthomonas albilineans*	CFBP 2523	*—*	Fiji
*Xanthomonas albilineans*	CFBP 1211	*—*	—
*Erwinia amylovora*	PGL Z1^a^	*Pyrus communis*	Italy
*Xanthomonas campestris* pv. *Campestris*	CFBP 1710	*Brassica oleracea* var. *botrytis*	France
*Pseudomonas syringae* pv. *Syringae*	CFBP 311	*Pyrus communis*	Indre et Loire-France
*Dickeya chrysanthemi* biovar *chrysanthemi*	CFBP 1346	*Chrysanthemum maximum*	Italy
*Pseudomonas savastanoi* pv *savastanoi*	CFBP 5050	*Olea europaea*	Portugal
*Agrobacterium vitis*	CFBP 2738	*Vitis vinifera*	Greece
*Agrobacterium larrymoorei*	CFBP 5473	*Ficus benjamina*	USA
*Agrobacterium rubi*	CFBP 5521	*Rubus* sp.	Germany
*Agrobacterium* sp. biovar 1	CFBP 5770	*Prunus persica*	Australia
*Agrobacterium* sp.	CFBP 2514	*Vitis vinifera*	Spain

CFBP: French Collection of Phytopathogenic Bacteria, Angers, France.

a Collection of CIHEAM-IAM, Bari, Italy.

### 2.10 *In planta* antagonistic activity of nisin against *Xylella fastidiosa*

**Figure 1:**
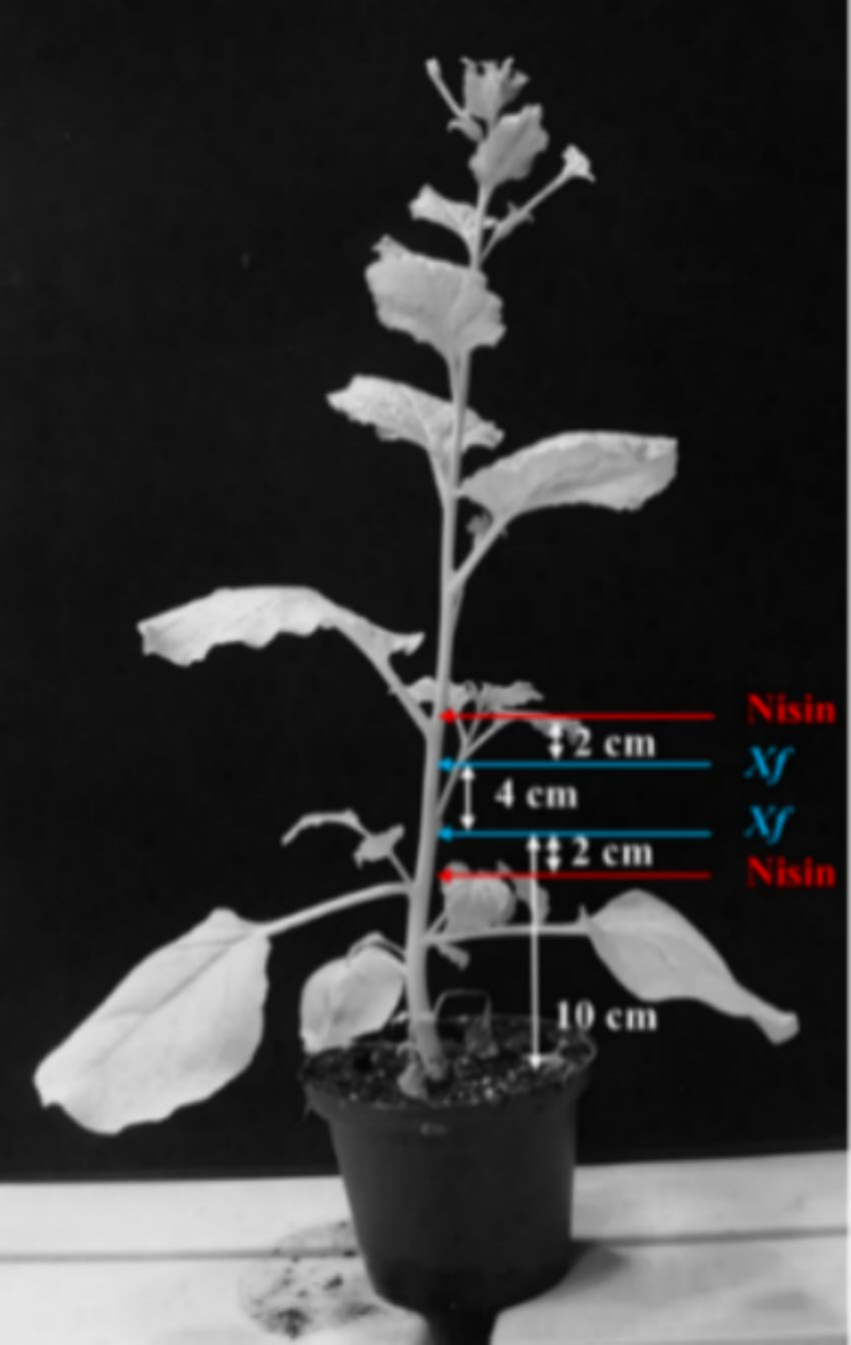
Schematic representation of *Xylella fastidiosa* and nisin inoculation sites.

To explore the antagonistic effect of nisin on *Xf* within plant tissues, an *in-planta* assay was conducted. This involved the *Xf* infection and the application of nisin to the stems of *Nicotiana benthamiana (N. benthamiana)*, which was validated as a model plant host for *Xf* (Baró et al., 2022). Briefly, one-month-old *N. benthamiana* plants were inoculated using a 0.1 mL insulin syringe, with the needle penetrating approximately half the plant stem diameter to access the vascular system. Two inoculations (25 μL each) of *Xf* suspension (OD_600_ = 0.32), prepared in PBS, were administered on the same side of the stem in a section of 4 cm at around 10 cm above the soil level. Two applications of 50 μL each of the nisin at 6 mg/mL (100 μL of nisin/plant, corresponding to 0.6 mg of nisin per plant) were performed at the same side of the stem in section of 8 cm at around 8 cm above the soil level (Figure 1). Preventive treatment with nisin was performed 24 h prior to *Xf* inoculation, whereas curative treatment was carried out 24 h after *Xf* inoculation. Each treatment consisted of 10 plants, including both positive and negative control groups, and inoculated plants were maintained in a controlled quarantine laboratory environment at 25°C. Two separate experiments were conducted to ensure the reliability of our findings.

Over a period of 74 days post inoculation (dpi), inoculated plants were visually inspected for the development of characteristic symptoms of *Xf* (i.e., leaf scorch), and movement of *Xf* within the plant was ascertained through TaqMan qPCR assay applied on genomic DNA extracted from leaves above the inoculation points (Figure 1). At the end of the experiment, leaves exhibiting suspected symptoms were analyzed for the presence of *Xf* using TaqMan qPCR assay as described earlier. Briefly, 1 g of leaf tissue was grinded with 2 mL of CTAB buffer, followed by heating at 65°C for 30 min. The plant extract was then centrifuged at 13,000 g for 15 min, and the resulting supernatant was washed with chloroform and precipitated in cold isopropyl alcohol. The resulting pellet, which contained the total DNA, was diluted in 100 μL of sterile water. Subsequently, 50 ng of this DNA was used for the TaqMan qPCR.

### 2.11 Stability and translocation of nisin in plants

**Figure 2:**
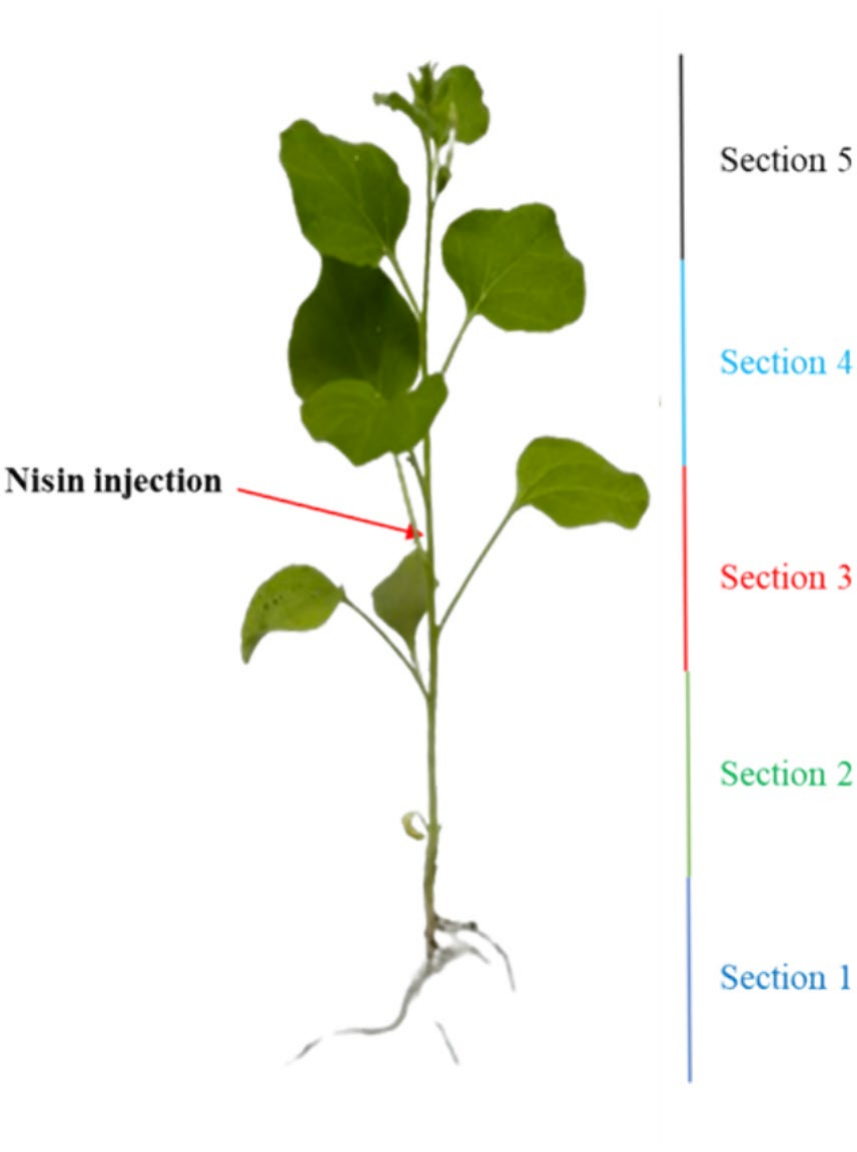
Illustration outlining the nisin injection and the segmentation of the plant into five distinct sections.

To explore the stability and translocation of nisin throughout the plant parts, one-month-old *N. benthamiana* plants (3 replicates) were inoculated as described in *in planta* section with 100 μL of nisin at 6 mg/mL. The plants were divided into five sections, with nisin inoculation carried out in the stem of section 3 (Figure 2). After 24 h, 48 h, 6 days, and 9 days, stems and leaves of each section were collected separately, ground in a mortar with 1 mL of sterile Milli-Q water, and then transferred to a centrifuge tube. After centrifugation at 3,000 g for 5 min at 5°C, the supernatant was filtered through a 0.22 μm nylon filter for RPLC-ESI-MS/MS analysis, using the same instrumentation as described above, with the following modifications. The gradient used for nisin quantification was: 0–2 min at 20% solvent B; 2–6 min linear from 20 to 85% (v/v) of solvent B; 6–9 min isocratic at 85% of solvent B; 9–10 min back to the initial composition, followed by 5 min equilibration time. For MS acquisitions, parameters were set as follows: sheath gas flow rate 35 arbitrary units (a.u.); auxiliary gas flow rate 5 a.u.; spray voltage 3.5 kV; capillary temperature 320°C; S-Lens radio frequency level 60 a.u. Acquisitions were performed in positive ion mode within the *m*/*z* interval 200–2000 and collisionally induced ionization (CID) energy was set as 35% a.u. for MS/MS ions fragmentation. Nisin was detected as tetra-charged [M + 4H]^4+^ or tri-charged [M + 3H]^3+^ ion, respectively, at *m*/*z* 839.3 and *m*/*z* 1118.7. Data were acquired in positive ionization with two transitions: 839.3 → 1080.7 and 1118.7 → 1451.7. The nisin standard A from *Lactococcus lactis* was used for calibration directly in plant sap to compensate the matrix effect exploring concentration from 6 μg/mL down to 0.06 ng/mL. The linearity of the method was determined by analyzing the standard plots associated with an 8-point standard calibration curve and the method was found to be linear in 3 orders of magnitude in the range 60–0.06 ng/mL. The LOD was calculated as 3 × the SD of the intercept or slope of the calibration curve, and LOQ was calculated as 10 × the SD of the intercept or slope of the calibration curve. The software Xcalibur 2.2 SP1.48 (Thermo Scientific) was used for raw RPLC-MS data.

## 3 Results

### 3.1 Antagonistic activity of *Lactococcus lactis* against *Xylella fastidiosa*

**Figure 3:**
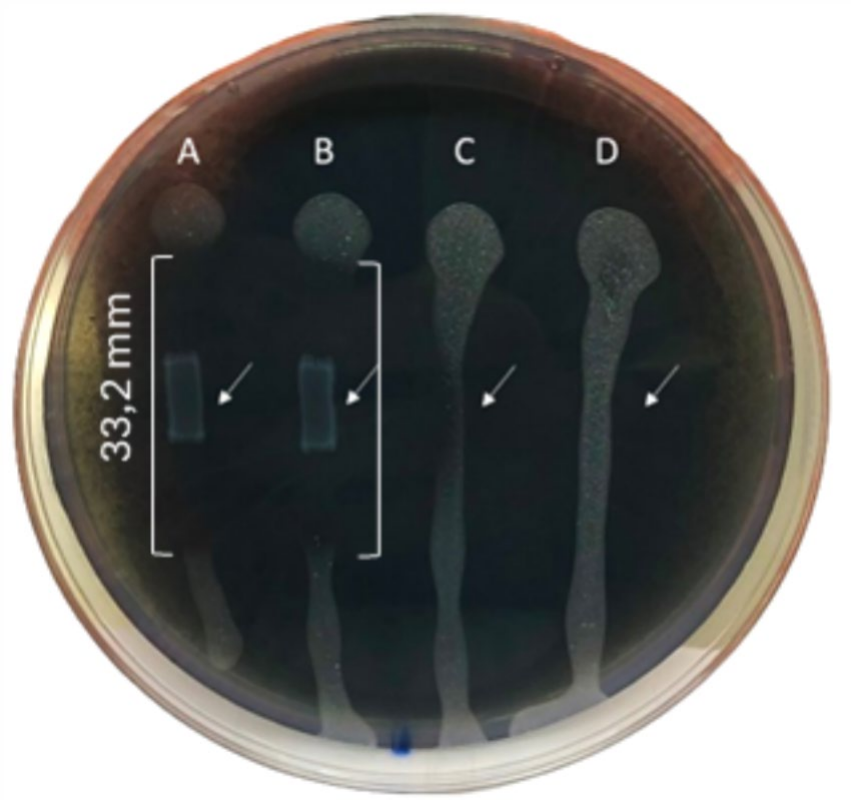
BYCE plate showing the antagonistic activity of *Lactococcus lactis* subsp. *lactis* against *Xylella fastidiosa* subsp. *pauca*. (A,B) *Xylella fastidiosa* challenged with *Lactococcus lactis* subsp. *lactis*. (C,D) *Xylella fastidiosa* treated with PBS. Brackets indicate the inhibition zones. Arrows indicate the sites of treatments.

The inhibitory activity of *L. lactis* against *Xf* growth was strong with an inhibition zone of 33.2 mm, highlighting the efficacy of *L. lactis* in restraining the *Xf* growth (Figure 3). In addition, *L. lactis* exhibited a lateral inhibition expansion, hindering the growth of *Xf* in the adjacent PBS-treated row C (Figure 3), demonstrating a high antagonism between *L. lactis* and *Xf*.

### 3.2 Characterization of *Lactococcus lactis*-secreted proteins

The RPLC-ESI-MS/MS analyses conducted on the inhibition zone, to investigate about the nature of compounds involved in the inhibitory activity of *L. lactis* against *Xf*, revealed the existence of nine peptides reported in Table 2. These peptides derive from different protein sources associated to plasma membrane or trans-membrane proteins of *L. lactis*. Some of these proteins are related to ATP-binding or hydrolysis activity, other to sugar, amino-sugar, and hexosamine metabolism (Table 2). The analyses could not reveal the presence of nisin in the inhibition zones, but the amino acid sequence of the transmembrane nisin-resistance protein was detected, which is a normally overexpressed by *L. lactis* to protect itself from the inhibitory effect of nisin. However, the RPLC-ESI-MS/MS has detected the presence of nisin, in a concentration of 345 ng/mL in the liquid medium after 24 h of incubation. This finding was taken as empirical evidence on the involvement of nisin in the inhibition of *Xf* growth. Based on this, we proceeded forward in the experimental design using pure nisin A in our research endeavors.

**Table 2:** List of peptides identified in the inhibition zone and their protein sources.

*m*/*z*	RT (min)	Peptide sequence	Protein accession	Protein name
604.9^2+^	9.0	KKIKKAPIAVL	P23648	Nisin-inhibitor protein
546.0^2+^	8.1	VAMLRSPLFG	Q9CJI9	ATP-dependent helicase/nuclease subunit A
546.0^2+^	10.8	LISSETAKEI	Q9CF53	Uronate isomerase
604.9^2+^	9.0	QKIVDLPIIGI	Q9CGC2	Putative N-acetylmannosamine-6-phosphate 2-epimerase
604.9^2+^	9.0	STLLQHLNGLL	Q9CIS8	Energy-coupling factor transporter ATP-binding protein
698.9^2+^	8.5	LSRESYTAELDL	Q9CGT6	Glutamine-fructose-6-phosphate aminotransferase
858.5^2+^	10.2	MVMAEDLAVRDNRIALL	A0AA42K6K3	Glycine-tRNA ligase subunit beta
858.5^2+^	10.2	VDTIQETLRIQADGR	Q9CFX8	Toxic anion resistance protein
1017.4^2+^	11.4	ELYSQEIELIAGIDEVGR	Q9CG17	Ribonuclease HII

*m*/*z*, mass-to-charge ratio; RT, retention time.

### 3.3 Determination of MLC of nisin

**Figure 4:**
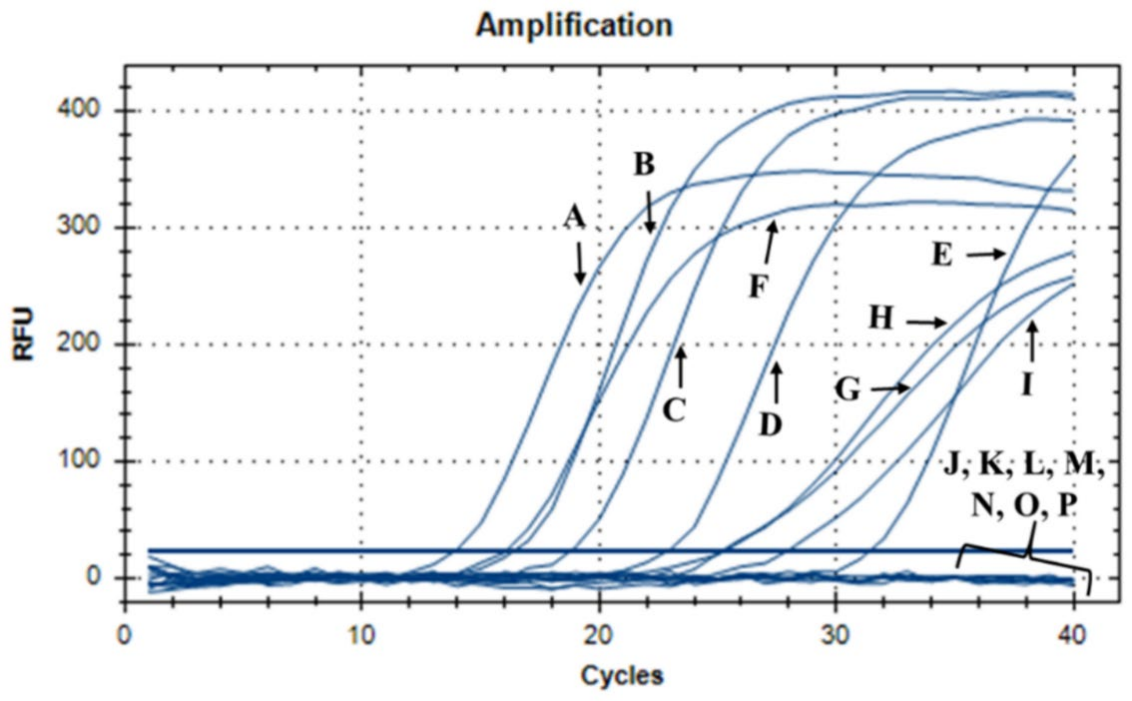
v-qPCR assay showing DNA amplification curves obtained from untreated *Xf*: (A) *Xf* (OD_600_ = 0.32), (B) *Xf* (OD_600_ = 0.16), (C) *Xf* (OD_600_ = 0.08), (D) *Xf* (OD_600_ = 0.04), (E) *Xf* (OD_600_ = 0.02), and from nisin-treated *Xf*: (F) nisin 0.05 mg/mL, (G) nisin 0.1 mg/mL, (H) nisin 0.2 mg/mL, (I) nisin 0.4 mg/mL, (J) nisin 0.6 mg/mL, (K) nisin 0.8 mg/mL, (L) nisin 1 mg/mL, (M) nisin 1.5 mg/mL, (N) nisin 3 mg/mL, (O) nisin 6 mg/mL. (P) Sterile distilled water used as negative control.

**Figure 5:**
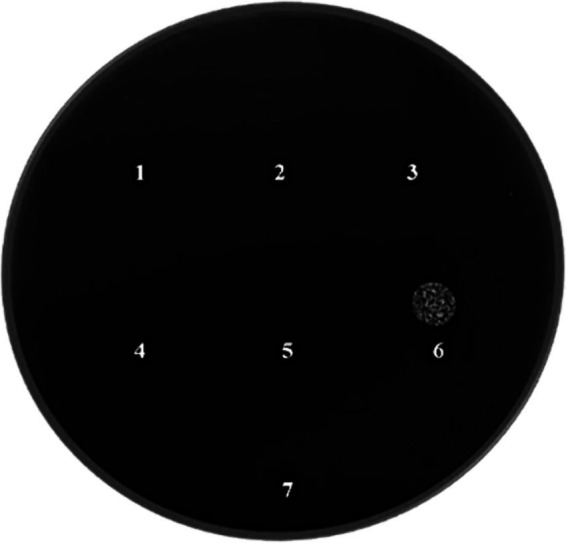
BCYE agar plate showing the spot assay of nisin against *Xf*; (1) nisin 3 mg/mL-treated *Xf*; (2) nisin 1.5 mg/mL-treated *Xf*; (3) nisin 1 mg/mL-treated *Xf*; (4) nisin 0.8 mg/mL-treated *Xf*; (5) nisin 0.6 mg/mL-treated *Xf*; (6) Untreated *Xf* used as a positive control; (7) PBS used as a negative control.

During this investigation, the antimicrobial efficacy of nisin against *Xf* was systematically assessed across different concentrations (6, 3, 1.5, 1, 0.8, 0.6, 0.4, 0.2, 0.1, 0.05 mg/mL). Employing v-qPCR as an initial *in vitro* screening method (Figure 4), the study aimed to determine the MLC of nisin for *Xf*. The obtained results were subsequently validated through a spot assay (Figure 5). According to the v-qPCR, nisin exhibited exceptional potency, effectively lysing *Xf* at different concentrations, with 0.6 mg/mL as the MLC. The spot assay results also supported this finding, showing the absence of *Xf* growth at concentration of nisin above 0.6 mg/mL.

### 3.4 Bactericidal activity of nisin against *Xylella fastidiosa*

**Figure 6:**
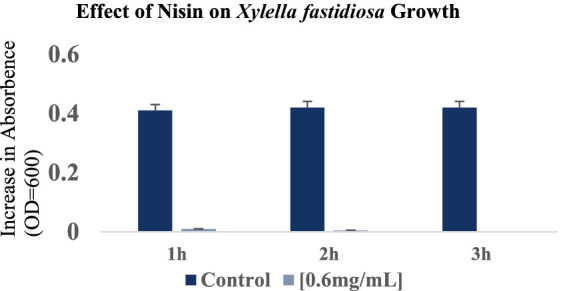
Optical density values at 600 nm showing the reduction in *Xylella fastidiosa* cell growth at 1 h, 2 h and 3 h after the addition of nisin at 0.6 mg/mL. The histograms show the mean values of three experimental repetitions with standard deviation.

**Figure 7:**
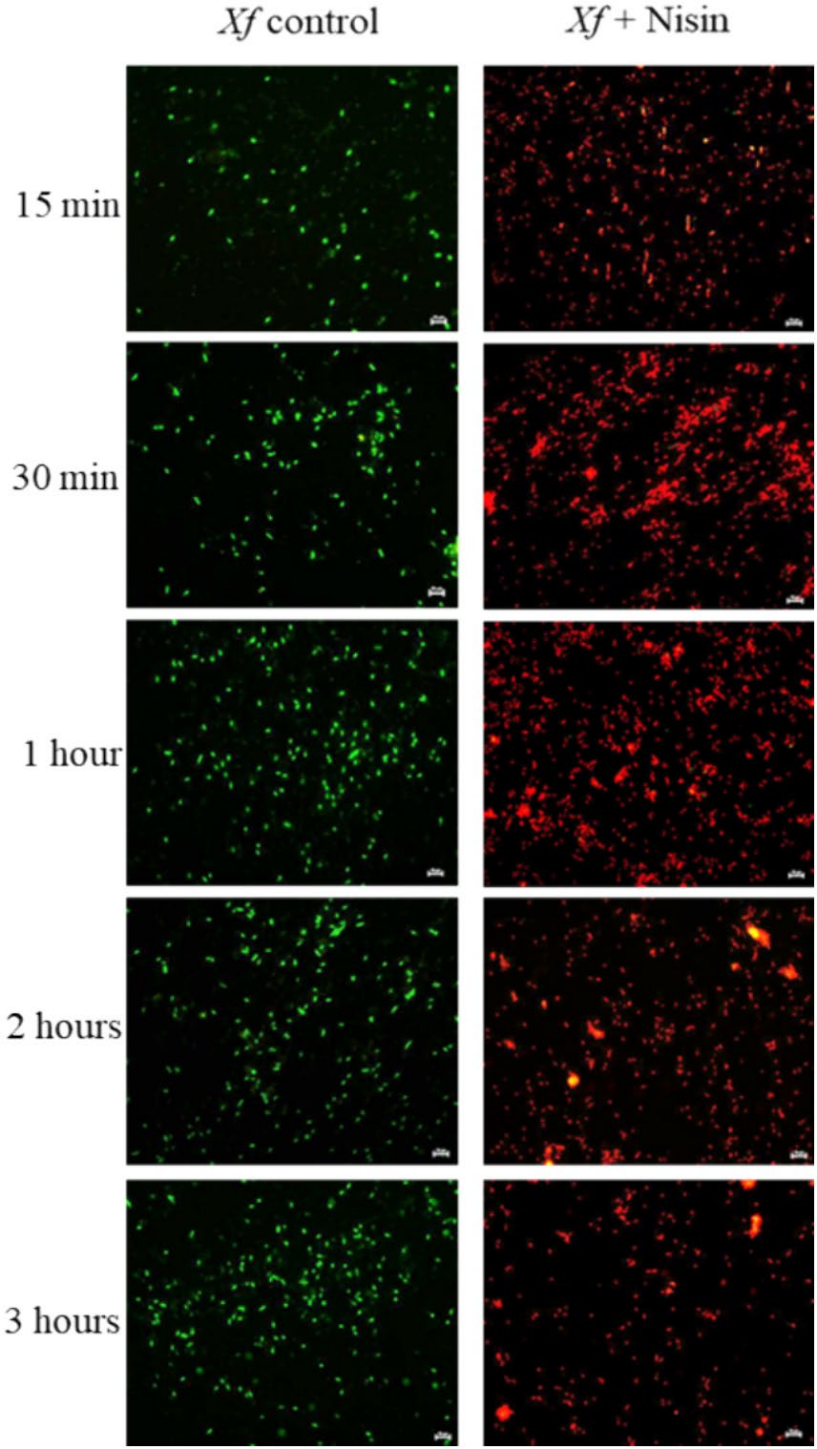
Fluorescent micrographs showing nisin-treated *Xylella fastidiosa* cells at 0.6 mg/mL. Green and red fluorescence represent viable and dead cells, respectively. Bar: 10 μm.

The response of *Xf* to nisin at 0.6 mg/mL was assessed during a 3 h incubation period. In summary, nisin showed almost complete reductive activity against *Xf* cells within the first hour of exposure, with a reduction of up to 90% (Figure 6). Whereas, this reductive activity endured for the entire 3 h post-contact period, resulting in the complete elimination of *Xf* cells (Figure 6). The efficacy of nisin against *Xf* cells was also investigated and confirmed by fluorescence microscopy (FM) using two dyes capable of distinguishing between live cells with intact plasma membranes (green channel) and dead bacteria with membranes compromised by nisin (red channel). The micrographs obtained corroborate the results of the turbidity assay, confirming once again that nisin exhibits significant and rapid destructive activity on *Xf*, as evidenced by the intense red channels, indicative of substantial lysing of *Xf* cells (Figure 7).

### 3.5 Effect of nisin on structural integrity of *Xylella fastidiosa*

**Figure 8:**
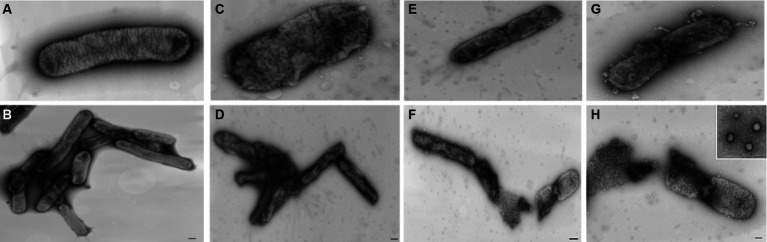
Transmission electron micrographs showing the impact of nisin on *Xf* cells. **(A,B)** Untreated *Xf* cells, used as control. **(C–H)** Nisin-treated *Xf* cells showing structural, cell wall, and cytoplasm alterations. Bar: 100 nm.

Analyzing the impact of nisin on *Xf* cells using TEM, micrographs disclosed remarkable alterations at the structural level, leading to fragmentation of the cell wall membrane (Figure 8G). Specifically, the nisin-treated *Xf* cells presented cytoplasmic condensation, fragmented cell walls, disrupted internal morphology, and alterations in the outer membrane structure; all resulting in destruction of both inner and outer bacterial membranes (Figure 8). Moreover, membranes of *Xf*-nisin treated cells showed serial pores and protrusions, accompanied by condensed cytoplasmic outflow from the outer membrane in the form of vesicles (Figure 8H).

### 3.6 Spectrum of action

For a sustainable use of nisin in an integrated pest management strategy, the antibacterial activity of nisin was tested against four *Xf* antagonistic bacteria (Table 1). The results showed that nisin had no antibacterial effect on them, suggesting its potential use, in combination with these antagonistic bacteria as part of integrated biocontrol approach against *Xf*. Similarly, nisin did not showed any effect on the 16 phytopathogenic bacteria tested, except for strains of *Xf* subsp. *pauca* (Table 1). This emphasizes the specific antibacterial activity of nisin at its MLC against *Xf* subsp. *pauca* among the tested bacteria.

### 3.7 Nisin effectiveness in controlling *Xylella fastidiosa in planta*

**Figure 9:**
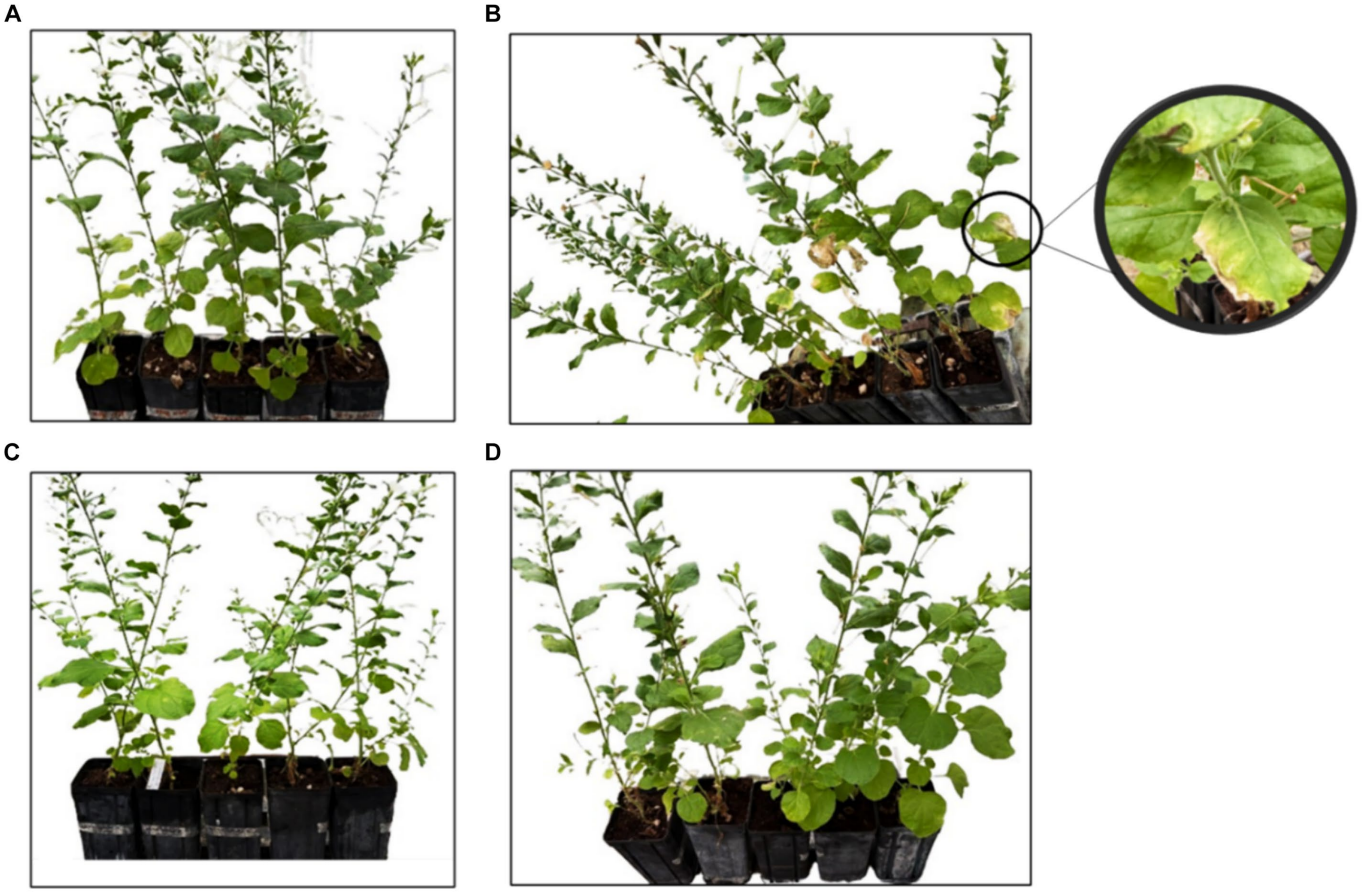
*In planta* assay showing the effect of nisin on *Xylella fastidiosa* subsp. *pauca* in *Nicotiana benthamiana* plants 74 dpi. **(A)** Healthy plants with no symptoms; **(B)** *Xf*-infected plants showing leaf scorch symptoms; **(C)** preventive nisin-treated *Xf*-infected plants with no symptoms; **(D)** curative nisin-treated *Xf*-infected plants with no symptoms.

**Figure 10:**
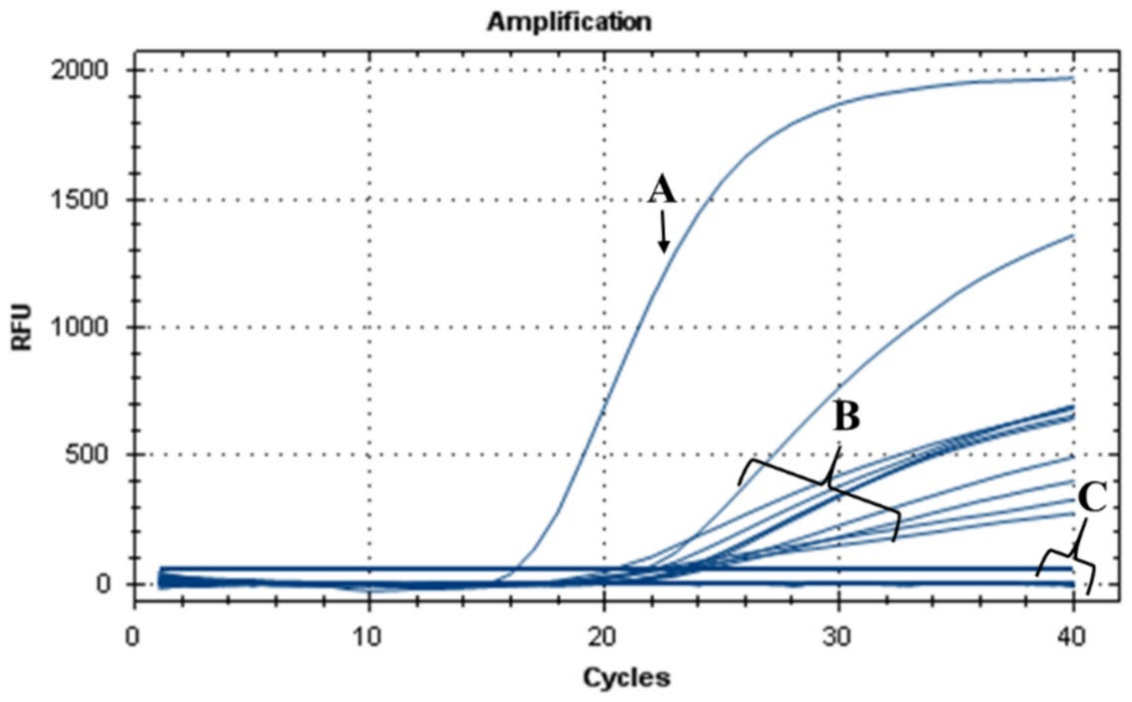
TaqMan-based qPCR assay showing DNA amplification curves obtained from (A) genomic DNA of *Xf* used as positive control, (B) genomic DNA extracted from leaves of nisin-untreated *Xf*-infected *Nicotiana benthamiana* plants (positive controls), (C) genomic DNA extracted from leaves of *Xf*-infected *Nicotiana benthamiana* plants treated preventively and curatively with nisin and from healthy plants used as a negative control.

The antibacterial effect of nisin against *Xf* in plant was evaluated in *N. benthamiana* plants treated with nisin before *Xf* infection (preventive) and after *Xf* infection (curative). Results of TaqMan-based qPCR performed 20 dpi on leaves collected above the inoculation sites, showed that *Xf* translocated only within the nisin-untreated infected plants (positive controls) (data not shown). By 40 dpi, the positive controls displayed typical *Xf* symptoms, i.e., leaf scorch (Figure 9B). In contrast, infected plants treated preventively and curatively with nisin demonstrated no typical symptoms of *Xf* (Figures 9C,D), indicating that nisin effectively countered the pathogen and prevented its impact on tobacco plants. At the end of the experiment (74 dpi), leaves with suspicious symptoms were analyzed for the presence of *Xf* using TaqMan-based qPCR assays, which showed the absence of the bacterium in all treatments; except leaves of positive controls (Figure 10).

### 3.8 *In planta* nisin detection using RPLC-ESI-MS/MS

**Figure 11:**
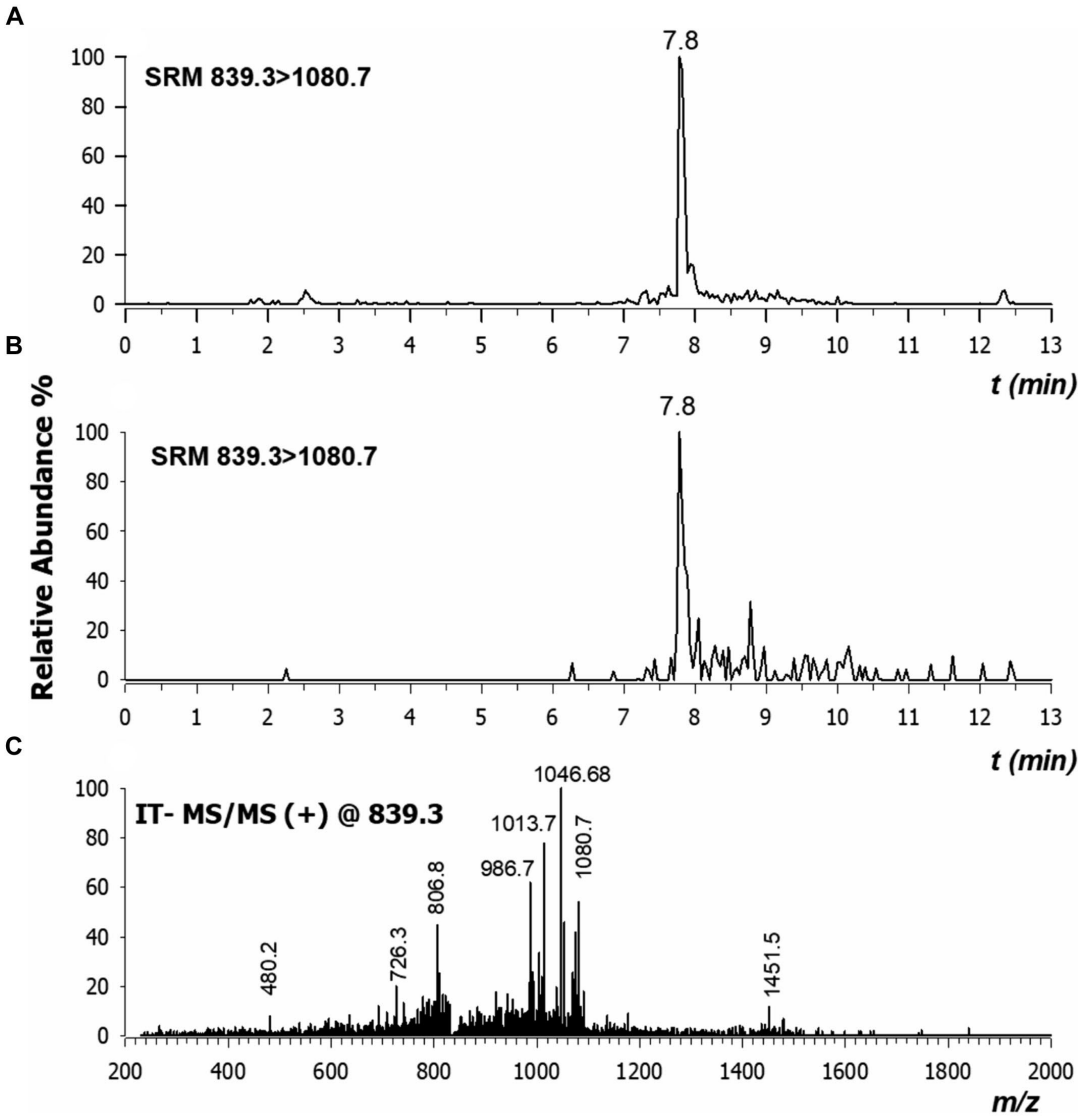
Chromatograms by RPLC-ESI-MS/MS in selected reaction monitoring (SRM) of nisin A in plant sap spiked at 30 ng/mL concentration **(A)** and in the stem of section 4 extracted after 24 h **(B)**. Major product ions detected in CID-MS/MS spectrum of tetra-charged ion at *m*/*z* 839.3 **(C)**. Values here reported are the means of three replicates.

To assess the stability and the translocation of nisin within *N. benthamiana*, RPLC-ESI-MS/MS analyses were carried out on sap recovered from different plant sections of three plants, as described above. Typically, low collisional energy and selected reaction monitoring (SRM) acquisition mode are employed in proteomic studies to enhance sensitivity and were thus also applied in the present case. When multi-charged molecules are analyzed, the transitions to a higher *m*/*z* ratio are considered highly. Therefore, the two most intense fragment ions with a higher *m*/*z* ratio than the precursor ions were chosen to develop the SRM method for nisin A. The highly specific transitions from the precursor ions [M + 4H]^4+^ and [M + 3H]^3+^ to higher *m*/*z* ratios were identified; the most intense one 839.3 → 1080.7 was used as quantifier while the other 1118.7 → 1451.7 was used as a confirmatory transition. As shown in Figure 11, a peak in the chromatogram appeared at a retention time of 7.8 min both in the standard spiked solution (A), and in a sample derived from stem (section 4) after 24 h (B). We also found that the tandem mass spectrum obtained in the stem of section 4 after 24 h (Figure 11C) had product ions identical to literature (Lavanant et al., 1998) and to those retrieved for standard solution (data not shown).

**Figure 12:**
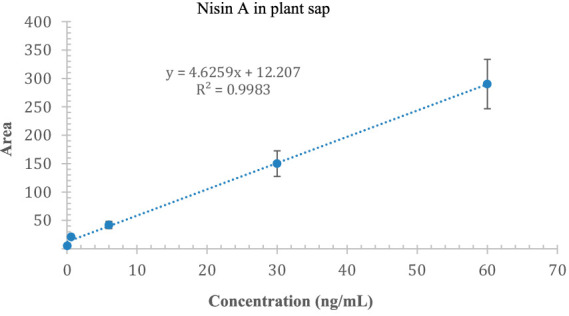
Calibration curve of nisin A in plant sap; the *x*-axis indicates the concentration of nisin, and the *y*-axis indicates the area of the SRM chromatogram.

We were able to qualitatively detect nisin A in stems, roots, and leaves; but to quantify the residual intact of nisin, we constructed an appropriate calibration curve spiking nisin A in blank plant sap. The linearity of the method was assessed at concentrations ranging from 0.06 to 60 ng/mL (5 levels) with a correlation coefficient of 0.9983 (Figure 12). The estimated LOD and LOQ values for nisin A were 2.1 and 7.1 ng/mL, respectively. Most of the values were below the LOD, some residual values above the LOD (2–4 ng/mL) were recovered in leaves (sections 3, 4 and 5) up to 9 days while the highest reliable concentration was found after 24 h in stem just above the injection point (section 4, 23.1 ng/mL). This experiment provides preliminary results on the stability of nisin in the plant, which can remain intact for up to 9 days after application, the period studied in our experiment.

## 4 Discussion

The emergence of *Xf* poses significant challenges to the agricultural sector worldwide, affecting economically significant crops and resulting in economic losses, changes in farming practices, and potential enduring damage to affected industries (Occhibove et al., 2020). To address the pressing need for effective solutions to combat *Xf* infections in many hosts, this study considered the use of lactic acid bacteria (LAB), which are recognized for their safety for human and animal consumption by the Food and Drug Administration (Ayivi et al., 2020), versatility across food, agriculture, and medicine. In particular, the use of nisin, a natural antimicrobial peptide (AMP) secreted by *L. lactis* subsp. *lactis*, to address *Xf* infections specifically highlights the potential of LAB in sustainable and environmentally friendly control strategies (Jaffar et al., 2023).

Our research has delimited the MLC of nisin, which at 0.6 mg/mL showed conspicuous antimicrobial potency across all *in vitro* assays, including spot assay, turbidity reduction assay, v-qPCR, FM, and TEM. Most importantly, FM and TEM results showed that nisin promptly reacts with *Xf* within the first 15 min of contact, leading to an immediate and complete lysis of the cellular membrane and with high potency. This observed effect aligns with the well-documented attributes of nisin, characterized by cationic and hydrophobic peptides (Musiejuk and Kafarski, 2023). The rapid and robust impact of nisin on *Xf* cells over a brief timeframe is most probably due to the high binding affinity of nisin to lipid II, disrupting the lipid bilayer. This causes the formation of pores, thereby enhancing membrane permeability and subsequent leakage of cellular contents, ultimately impeding bacterial growth (Meade et al., 2020). This action is most likely accentuated by the nature of *Xf* in forming biofilm-like aggregates, for which the transition from the planktonic to the biofilm-associated state involves profound physiological changes including changes in the protein composition of the bacterial cell envelope. Indeed, changes in outer membrane protein composition are known to have a profound effect on the sensitivity of Gram-negative bacteria to detergents, antibiotics, and bacteriophages (Nikaido, 1996). However, the composition and structure of the outer membrane of *Xf* is still not entirely understood; and more studies are needed to depict the mechanism of nisin in affecting the *Xf* cells.

To extend the knowledge on nisin’s antibacterial activity and its stability in a complex environmental context, serial experiments were performed in *Xf-*host plant model, i.e., *N. benthamiana*. The results consistently showed that nisin was able to prevent the development of *Xf* symptoms over a period of 74 days and to exert antibacterial activity despite the presence of plant compounds capable of degrading nisin, i.e., proteases. Indeed, nisin remained intact within the plant up to 9 days after injection, justifying the nisin efficacy *in planta* against *Xf* in both preventive and curative applications. This attribute, coupled with its strong and rapid antibacterial activity, its selectivity for *Xf* cells, as well as its effortless translocation, makes it an indispensable option in tackling the *Xf* infections.

Top of form being a naturally occurring antimicrobial peptide, nisin has several advantages over synthetic antimicrobial peptides, which may be produced at a considerable expense and require certain conditions for synthesis and purification. It is produced by *L. lactis* in a straightforward laboratory setting involving common media and incubation conditions (Khelissa et al., 2021). In our laboratory conditions, nisin was secreted by *L. lactis* subsp. *lactis* strain ATCC 11454 at a concentration of 345 ng/mL in YPG liquid medium after 24 h of incubation. This strain is also reported in the literature to produce 0.2 g/L of nisin in 25% milk and 25% MRS medium (Penna et al., 2005). However, the presence of the nisin in the inhibition zone was not discernable, most likely due to the difficulty of tracking nisin on solid media, contrarily to the liquid culture media. The ease of producing large quantities of nisin in the laboratory and at low cost makes this compound a sustainable approach for agricultural application in the field. Unlike in the medical and food preservation domains, substantial nisin purification which might lead to increased costs, is not required in agriculture. On a practical level, the application of nisin in the field can be done by trunk injection methods; as in our case *Xf* is a xylem-limited pathogen difficult to reach, the spraying method is ineffective and inapplicable. Indeed, the trunk injection approach has been used to control fire blight bacterial disease of apple trees and huanglongbing of citrus caused by the systemic pathogen *Candidatus liberibacter* Garnier (Aćimović et al., 2014; Puttamuk et al., 2014). Furthermore, the same bacteria that produce nisin are also capable of creating a complex of substances that might support soil enrichment and plant development, in addition to its antibacterial qualities (Naskar and Kim, 2021; Raman et al., 2022). This dual functionality could present an integrated strategy for both treating and fortifying plants in their supposed susceptibility to *Xf*.

In conclusion, we are confident that nisin represents a noteworthy sustainable and practical tool to treat challenging *Xf* subsp. *pauca* infections in plants. This aligns with the principles of greener agriculture, in accordance with European Union regulations on quarantine pests, particularly for *Xf*. Indeed, bacteriocin-based biobactericides are gaining more attention for their compatibility with eco-friendly and sustainable agricultural practices. They present a realistic approach for controlling plant pathogenic bacteria, replacing, or complementing the existing agrochemicals within comprehensive control programs. Lastly, further studies will be important to elucidate the ability of nisin and nisin-producing bacteria to enhance plant resistance to *Xf* subsp. *pauca* and to promote better growth and development of *Xf* subsp. *pauca* host plants in agricultural production. Further research is needed to explore nisin potential applications in field conditions against different *Xf*-host plant species.

## Data availability statement

The raw data supporting the conclusions of this article will be made available by the authors, without undue reservation.

## Author contributions

MS: Conceptualization, Data curation, Formal analysis, Investigation, Methodology, Software, Validation, Visualization, Writing – original draft, Writing – review & editing. KH: Conceptualization, Data curation, Formal analysis, Investigation, Methodology, Software, Validation, Visualization, Writing – original draft, Writing – review & editing. FV: Data curation, Investigation, Validation, Visualization, Writing – review & editing. AS: Data curation, Investigation, Validation, Visualization, Writing – review & editing, Formal analysis, Methodology, Software. OC: Data curation, Formal analysis, Investigation, Software, Visualization, Writing – review & editing. CC: Data curation, Formal analysis, Investigation, Visualization, Writing – review & editing, Methodology, Validation. MB: Formal analysis, Visualization, Writing – review & editing, Software. AT: Formal analysis, Software, Visualization, Writing – review & editing. TE: Formal analysis, Software, Visualization, Writing – review & editing, Conceptualization, Data curation, Funding acquisition, Investigation, Methodology, Project administration, Resources, Supervision, Validation, Writing – original draft.
